# Primary care doctors' practices in managing menopausal symptoms and views on patient clinical conditions influencing their decision to offer menopause hormone therapy: A cross-sectional study

**DOI:** 10.51866/oa.843

**Published:** 2025-03-14

**Authors:** Tiong Lim Low, Ai Theng Cheong, Navin Kumar Devaraj, Rohayah Ismail

**Affiliations:** 1 MD, MMed (Fam Med), Klinik Kesihatan Jinjang, 2, Jalan Jinjang Setia 3, Jinjang Utara Tambahan, Kepong, Kuala Lumpur, Malaysia. Email: ivzh0120@gmail.com; 2 MBBS (UM), MMed (Family Medicine) (UM), PhD (UM), Department of Family Medicine, Faculty of Medicine and Health Sciences, Universiti Putra Malaysia Serdang, Selangor, Malalysia. Email: cheaitheng@upm.edu.my; 3 MBBS, MMed (Fam Med), Department of Family Medicine, Faculty of Medicine and Health Sciences, Universiti Putra Malaysia, Serdang, Selangor, Malaysia.; 4 MD, MMed (Fam Med), Klinik Kesihatan Sentul, 29, Jalan 3/48a, Bandar Baru Sentul, Sentul, Kuala Lumpur, Malaysia.

**Keywords:** Primary healthcare, Menopause, Oestrogen hormone therapy, Practice guideline

## Abstract

**Introduction:**

Menopausal symptoms, such as vasomotor disturbances, mood changes and atrophic vaginitis, significantly affect women’s quality of life. This study aimed to examine how primary care doctors (PCDs) manage these symptoms and the clinical conditions influencing their decision to offer menopause hormone therapy (MHT), bridging gaps between guidelines and real-world practices.

**Methods:**

A cross-sectional study was conducted from June to October 2022 among PCDs in public health clinics in Selangor, Kuala Lumpur and Putrajaya. An online survey was distributed to 1301 PCDs, achieving a 42.9% response rate (559 respondents). Data were analysed descriptively.

**Results:**

Lifestyle modifications were the most common recommendation (98.4%), while complementary treatments were widely recommended (54.8%). MHT was discussed by 83.5% of the PCDs but directly prescribed by only 0.9%, with 66.0% referring patients to tertiary care. MHT was primarily offered for vasomotor symptoms (80.5%) and mood disorders (56.7%) but less commonly for non-communicable diseases such as hypertension (14.1%) and diabetes mellitus (25.2%). It was withheld in cases of breast cancer (91.9%) or venous thromboembolism (86.0%), with breast cancer concerns being a major barrier (75.3%).

**Conclusion:**

Lifestyle modifications and complementary treatments were common practice options. MHT discussions were frequent, but prescription was limited due to patient concerns and comorbidities. Targeted updates to guidelines and tools can support healthcare professionals in counselling, risk assessment and effective management of menopausal symptoms.

## Introduction

Menopausal symptoms can present in various forms, including vasomotor symptoms (e.g. hot flashes and night sweats), atrophic vaginitis, mood disturbances, sleep disorders and decreased libido. These symptoms can significantly affect a woman’s quality of life. Different approaches are available to manage these symptoms, ranging from menopausal hormone therapy (MHT) to supplements and complementary treatments. Nevertheless, MHT remains the recommended treatment for menopausal symptoms, particularly for managing vasomotor symptoms.^1,2^ However, local studies have shown that many patients prefer to use vitamins and supplements, despite the lack of strong evidence supporting their efficacy.^3,4^ Patients often seek medical attention only when their symptoms start to affect their daily lives, and primary care clinics are typically their first points of contact.^4^ On average, primary care doctors (PCDs) may see one to two patients with menopausal symptoms per month,^5^ although this number could be underreported due to variations in symptom severity and patient awareness.

A previous study focused on the prevalence of offering MHT among PCDs and determined its associated factors, including PCDs’ sociodemographic data, training, attitudes, selfperceived knowledge, availability of MHT and perceived barriers.^5^ Conversely, this study aimed to examine the practices of PCDs in managing menopausal symptoms and explore their views on clinical conditions influencing their decision to offer MHT. This is anticipated to provide another perspective from the previous article, particularly regarding real-world practices in managing menopause, and highlight gaps between guidelines and practices.

## Methods

This study is part of a larger investigation examining the prevalence of MHT and its associated factors among PCDs.^5^ A cross-sectional study was conducted from 1 June to 31 October 2022. The study population included PCDs practising in public health clinics in Selangor, Kuala Lumpur and Putrajaya selected using universal sampling. The inclusion criteria were registration with the Malaysian Medical Council, a valid Annual Practice Certificate and at least 12 months of primary care experience. PCDs not treating patients at least once a week were excluded.

Ethical approval was obtained from the Medical Research and Ethics Committee, Ministry of Health, Malaysia, and permission to conduct the study was granted by state and district health authorities. A list of primary health clinics (22 clinics under Kuala Lumpur and Putrajaya and 81 clinics under Selangor) was obtained from Unit Primer under the state health offices. The contacts of the doctors in charge of the clinics were obtained through communication with the district health officers. The number of PCDs working in the respective clinics was obtained through the doctors in charge of the clinics. The total number of potentially eligible PCDs was found to be 1301. For recruitment of participants in the study, an advertisement of invitation for participating in the survey, the approval letter from the state and the link for the online survey (Google Forms) were sent through the WhatsApp accounts of the doctors in charge. The doctors in charge then distributed the online survey to all PCDs in their clinics and were given reminders twice a month (up to six times during the survey period) to circulate the survey to eligible respondents.

The tool used in this study was a questionnaire adapted from an Australian study, designed to explore healthcare professionals’ practices in managing menopause.^6^ Adaptations were made to better suit the local context. Content validation was performed by three senior family medicine specialists. Thereafter, a pilot study was conducted with 30 healthcare professionals, who were excluded from the final sample. The pilot study provided face validity, wherein participants offered feedback. Such feedback led to minor corrections, focusing on grammar and wording, to enhance clarity. We did not proceed with statistical testing such as reliability testing and factor analysis, as the questionnaire items were analysed individually as categorical data without composite scoring.

In this study, several key outcomes were evaluated including the following: the prevalence of different practices by PCDs in treating symptomatic menopausal women as well as the views of PCDs on patient clinical conditions and age influencing their decision to offer MHT or not. All of these outcomes were descriptively analysed, with the results expressed in frequencies and percentages.

## Results

This study achieved a response rate of 42.9% (559/1301). The majority of the respondents were women (77.8%) and were working as medical officers (89.1%) (Table 1). The median age was 36 years (Interquartile range=6).

**Table 1:** PCDs’ sociodemographic characteristics (N=559).

Characteristic		Frequency	Percentage
	<35	154	27.5
Age, year	35-39	267	47.8
	≥40	138	24.7
Sex	Male	124	22.2
	Female	435	77.8
	Malay	316	56.5
Ethnicity	Chinese	108	19.3
	Indian	113	20.2
	Other	22	4.0
Position	Medical officer	498	89.1
	Family medicine specialist	61	10.9
Years of practice in primary care settings	<5	169	30.2
	5-9	209	37.4
	≥10	181	32.4

Lifestyle modifications were the most often recommended treatment modality for symptomatic menopausal women, with nearly all respondents endorsing them (98.4%). Other common approaches included discussions on MHT (83.5%) and the use of complementary and alternative therapies (54.8%), while non-hormone treatments such as selective serotonin reuptake inhibitors (SSRIs) and gabapentin were less commonly used (15.4%) (Table 2). Although 66.9% of the respondents offered MHT, only 0.9% directly prescribed it, with the majority (66.0%) referring patients to tertiary care for MHT initiation.

**Table 2:** Prevalence of different practices by PCDs in treating symptomatic menopausal women (N=559).

Practice	Frequency	Percentage
Lifestyle modifications^†^	550	98.4
Discussion on MHT	467	83.5
Offering of MHT	374	66.9
• Prescription of MHT	5	0.9
• Referral of patients to tertiary care centres for MHT initiation	369	66.0
Complementary and alternative therapies^*^	273	54.8
Cognitive behaviour therapy	120	21.5
Non-hormone therapy (SSRI or gabapentin)	86	15.4

† Lifestyle modifications: Encourage regular exercise, adequate sleep and a balanced diet.

* Complementary and alternative therapies: Suggest herbal treatment, massage, acupuncture, yoga or meditation.

The respondents most frequently cited vasomotor symptoms as the condition warranting the offer of MHT (80.5%), followed by mood disorders (56.7%) and osteoporosis (49.0%) (Table 3). Reduced libido (48.1%) and atrophic vaginitis (34.5%) were less commonly identified as reasons for offering MHT.

**Table 3:** PCDs’ views on patient clinical conditions influencing their decision to offer MHT (N=559).

Patient clinical condition	Frequency	Percentage
Vasomotor symptom	450	80.5
Mood disorder (anxiety/depression)	317	56.7
Osteoporosis	274	49.0
Reduced libido	269	48.1
Atrophic vaginitis	193	34.5
Dyspareunia	181	32.4
Incontinence	144	25.8
Muscular skeletal pain/lethargy	129	23.1

MHT was predominantly not offered to patients with a history of breast cancer (91.9%) or venous thromboembolism (VTE) (86.0%) (Table 4). Other frequently cited reasons for withholding MHT included poorly controlled hypertension (85.0%) and diabetes mellitus (70.3%).

**Table 4:** PCDs’ views on patient clinical conditions influencing their decision not to offer MHT (N=559).

Patient clinical condition	Frequency	Percentage
History of breast cancer	514	91.9
History of VTE	481	86.0
Hypertension (poorly controlled)	475	85.0
History of uterine cancer (in remission)	458	81.9
History of atherosclerotic disease	438	78.4
Concern about breast cancer	421	75.3
Diabetes mellitus (poorly controlled)	393	70.3
Dyslipidaemia (poorly controlled)	354	63.3
Family history of breast cancer	348	62.2
Obesity	269	48.1
Diabetes mellitus (well-controlled)	141	25.2
Hypertension (well-controlled)	79	14.1
Dyslipidaemia (well-controlled)	72	12.9

The majority of the respondents considered MHT appropriate for women aged 50-55 years (85.6%) and 56-60 years (67.6%), with many offering it to women aged 40-49 years (61.7%) (Table 5). Fewer respondents recommended MHT for women under 40 years (25.2%) or over 60 years (19.5%).

**Table 5:** PCDs’ views on patient age influencing their decision to offer MHT (N=559).

Patient age, year	Frequency	Percentage
<40	141	25.2
40-49	345	61.7
50-55	479	85.6
56-60	378	67.6
>60	109	19.5

## Discussion

We found that almost all PCDs advised their patients on lifestyle modifications to alleviate menopausal symptoms. This approach is likely influenced by the widespread acceptability of lifestyle modifications, which are perceived by both healthcare professionals and patients as interventions with minimal or no side effects. Furthermore, healthy lifestyles contribute to overall well-being, although evidence supporting specific measures, such as exercise for reducing hot flushes, remains limited.^7^

A noticeable contrast exists between PCD recommendations and patient preferences for managing menopausal symptoms. While most PCDs (66.9%) in our study offered MHT, it remains a less favoured option among patients, as highlighted in both local and international studies.^3,8,9^ For instance, a local study of 258 Malaysian women found that only 20.3% used MHT, despite 53.4% reporting that menopausal symptoms affected their quality of life.^3^ Similarly, a study involving 4014 working women in the United Kingdom revealed that only 14% used MHT, with 10% leaving their jobs due to difficulties coping with menopausal symptoms.^8^ The same survey also found that 28% of patients offered MHT expressed concerns based on information provided by healthcare professionals, which made them wary of its use.^8^ Patients also cited fears of side effects and concerns about ‘using synthetic stuff as barriers to MHT uptake.^9^ These findings underscore the critical need for effective communication to address patient apprehensions and misconceptions.

From discussing MHT to offering it, PCDs consider patient clinical conditions to determine its suitability. Our study revealed that 80.5% of the PCDs agreed to initiate MHT for patients with vasomotor symptoms, consistent with evidence supporting MHT’s effectiveness in relieving these symptoms.^10^ For other clinical indications, MHT is also advocated by local and international guidelines for managing osteoporosis.^1,2^ However, only 49% of the PCDs in our study offered MHT for osteoporosis, echoing findings from an Australian study where MHT was underprescribed for bone health.^11^ In that study, healthcare professionals often referred patients to endocrinologists, citing uncertainties about efficacy and concerns over potential side effects.^11^ This hesitancy may limit access to an effective treatment for osteoporosis among menopausal women.

Herein, 56.7% of the PCDs were willing to offer MHT for mood disorders, acknowledging the significant impact of menopausal symptoms on quality of life. However, the use of MHT for mood disorders remains an area requiring further evidence to support its indication.^12^

Nearly half of the PCDs in our study were hesitant to offer MHT to patients with well-controlled non-communicable diseases (NCDs) such as hypertension, dyslipidaemia, diabetes mellitus and obesity. This caution may stem from concerns about cardiovascular risks, despite evidence showing that MHT can reduce cardiovascular risks if started before the age of 60 years or within 10 years of menopause. A meta-analysis of 19 randomised controlled trials involving over 40,000 postmenopausal women found no significant increase in all-cause mortality, cardiovascular death or myocardial infarction.^13^ Additionally, starting MHT within 10 years of menopause was associated with reduced mortality (Relative Risk=0.70, 95% CI=0.52-0.95) and fewer cardiac events (Relative Risk=0.52, 95% CI=0.29-0.96).^13^ Reluctance to offer MHT in this group may reflect confusion about current evidence or limited training, as highlighted in earlier findings.^5^ Addressing this through updated guidelines and practical toolkits could support better decision-making. A useful tool, The Practitioner’s Toolkit for Managing the Menopause, has been developed and revised in accordance with the latest evidence and endorsed by the International Menopause Society to facilitate risk assessment and counselling for MHT, serving as a potential model for local adaptations.^14^

Concerns about VTE also influenced PCD decisions, as VTE risk is the second-highest barrier preventing PCDs from offering MHT. Transdermal MHT formulations, which observational studies have shown to carry lower VTE risks compared to oral MHT formulations, offer a safer alternative.^15^ However, limited access due to high costs and exclusion from the Ministry of Health formulary poses significant challenges.^16^

The fear of breast cancer significantly hindered MHT recommendations, with 75% of the PCDs citing it as a major concern. The risk of breast cancer with MHT is complex, varying by formulation, timing and duration, which has caused confusion among PCDs.^17^ Patients shared similar apprehensions, frequently associating MHT with an increased risk of breast cancer.^9^ This mutual fear likely reinforces negative attitudes towards MHT, contributing to treatment delays or avoidance. Consequently, many PCDs felt more comfortable recommending complementary and alternative therapies, a trend observed globally.^11,18^ However, many alternative treatments lack robust evidence and may offer limited benefit.

The timing of MHT initiation plays a critical role in determining cardiovascular risks.^13^ Our study found that 20% of the PCDs were willing to offer MHT to patients over 60 years old, despite general recommendations against it due to increased cardiovascular risks.^1,2^ This again reflects insufficient training, as reported in a previous study, where many PCDs cited a lack of expertise in menopausal management.^5^ Conversely, only a small proportion of the PCDs offered MHT to patients under 40 years old. The outcome of this approach remains unclear, as our study did not explore how PCDs manage patients presenting with premature menopause. One possibility is that such patients are referred to hospitals for further investigation of the underlying cause, as premature menopause is relatively uncommon, rather than initiating treatment in the primary care setting. Alternatively, there is a risk that these patients’ symptoms may be overlooked and left untreated. Further research is needed to better understand and address these gaps in management.

There are several limitations of this study. First, the study was conducted exclusively in public health clinics; thus, the outcomes may not fully represent practices in private clinics, which also play a significant role in the country’s primary care landscape. Second, the study employed a convenience sampling method and relied on descriptive analysis, limiting its ability to establish cause-and-effect relationships between the studied factors. However, the findings could provide some insight into gaps between guidelines, evidence and real-world practices in managing menopausal symptoms in primary care settings.

## Conclusion

Our study highlights the range of treatment modalities used by PCDs to manage menopausal symptoms, with lifestyle modifications being the most common. While discussions about MHT are frequent, converting these into actual prescriptions remains a challenge. MHT is often offered for vasomotor symptoms and mood-related issues, but there is hesitation in prescribing it for osteoporosis and common NCDs such as hypertension and diabetes mellitus. Additionally, patient concerns about breast cancer risks continue to be a significant barrier to MHT use. These findings suggest the need for targeted updates to current guidelines as well as the development of local toolkits or algorithms to facilitate patient risk assessment and counselling for MHT.

## Data sharing statement

Raw data are available only upon request with permission from the Ministry of Health, Malaysia.

**How does this paper make a difference in general practice?**

- The study highlights gaps between guidelines, evidence and real-world practices in managing menopausal symptoms in primary care settings.
- Menopausal hormone therapy prescription is complex and individualised, influenced by patient concerns, comorbidities and other factors.
